# The prevalence and characteristics of microalbuminuria in the general population: a cross-sectional study

**DOI:** 10.1186/1756-0500-6-256

**Published:** 2013-07-07

**Authors:** Satoru Tanaka, Hiroyuki Takase, Yasuaki Dohi, Genjiro Kimura

**Affiliations:** 1 Department of Cardio-Renal Medicine and Hypertension, Nagoya City University Graduate School of Medical Sciences, Nagoya, Japan; 2 Department of Internal Medicine, Enshu Hospital, Hamamatsu, Japan; 3 Department of Internal Medicine, Asahi Rousai Hospital, Owariasahi, Japan

**Keywords:** Blood pressure, General population, Hypertension, Microalbuminuria, Salt

## Abstract

**Background:**

Microalbuminuria is a marker of cardiovascular events. This study investigated the prevalence of microalbuminuria in the general population and the factors that can affect urinary excretion of albumin.

**Methods:**

Apparently healthy subjects who participated in a health checkup at our hospital were enrolled in this study (n = 7963, male 64.0%, 56.2 ± 11.8 years old) Urine samples were collected for the measurement of albumin concentrations, which were expressed as the ratio of urinary albumin to creatinine concentrations (UACR [mg/g Cr]). Individual salt intake was assessed by estimating the 24-hour urinary salt excretion of subjects.

**Results:**

The mean blood pressure was 124 ± 15/76 ± 10 mmHg and 31.6%, 7.4%, and 44.1% of subjects had hypertension, diabetes mellitus, and dyslipidemia, respectively. Urinary albumin was detected in 7265 subjects (91.2%: UACR ≥ 300 mg/g Cr, 0.5%; 300 > UACR ≥ 30 mg/g Cr, 4.6%; 30 > UACR ≥ 20 mg/g Cr, 2.4%; 20 > UACR ≥ 10 mg/g Cr, 8.7%; 10 > UACR ≥ 5 mg/g Cr, 21.8%; UACR < 5 mg/g Cr, 53.2%). In subjects with detectable albuminuria, UACR was independently correlated with age, systolic blood pressure, serum creatinine, fasting plasma glucose, and salt intake after adjustment for possible factors (*P* < 0.0001).

**Conclusion:**

The prevalence of microalbuminuria was found to be 4.6% in the general population. The urinary excretion of albumin was closely associated with blood pressure and salt intake. These data indicated the importance of salt restriction for the prevention of cardiovascular disease and end-stage renal disease.

## Background

Recent studies have established that microalbuminuria is an important cardiovascular risk factor [1,2]. Clinical data have demonstrated that left ventricular hypertrophy, increased intima-media thickness of the carotid artery, and subclinical cardiovascular diseases are associated with microalbuminuria among individuals at high risk for cardiovascular disease [3-5]. Furthermore, epidemiological and experimental studies have established that high levels of urinary albumin excretion are associated with an increased incidence of cardiovascular mortality as well as mortality from all causes [6]. Most of these data came from observations involving high-risk patients. The association between urinary albumin levels and increased cardiovascular mortality in high-risk patients is evident even at urinary albumin levels below the clinically defined threshold for microalbuminuria.

Urinary albumin excretion is also a predictor of mortality from all causes in the general population [7]. The excess risk is more attributable to death from cardiovascular causes and this relationship is already apparent at ‘normal’ levels of albuminuria [3,8]. The establishment of an association between urinary excretion of albumin and cardiovascular morbidity and mortality in the general population may support the measurement of urinary albumin as a cardiovascular disease marker. This would enable the identification of individuals who may benefit from aggressive risk reduction for the primary prevention of cardiovascular disease. Thus, the present study investigated the range of urinary excretion levels for albumin and the prevalence of microalbuminuria. Through these data we sought to identify factors that may affect urinary excretion of albumin in the general population.

## Methods

The study was performed in accordance with the principles of the Declaration of Helsinki, and the Ethics Committee of Enshu Hospital approved the study protocol. Written informed consent was obtained from all subjects prior to the start of the study.

### Study settings and subjects

A total of 8155 persons who visited our hospital for a yearly physical checkup from April 2010 to March 2011 were screened for eligibility to be included in the present study. Subjects with incomplete data were excluded and the rest of the 7963 subjects were finally included in the present study. In addition to the routine examination of subjects in our health checkup program (physical examination, chest X-ray, electrocardiogram [ECG], and laboratory assessment of cardiovascular risk factors), urine was sampled for the measurement of urinary albumin in the morning after an overnight fast. Cross-sectional analyses were performed in order to investigate the prevalence of albuminuria and factors closely related to the urinary excretion of albumin.

Urinary albumin concentrations were measured by a turbidimetric immunoassay (analytical range ≥ 1.1 mg/L) and are expressed as the ratio of concentrations of urinary albumin to urinary creatinine (UACR [mg/g Cr]). UACR was recorded as 0 mg/g Cr in those subjects with a urinary albumin concentration below the analytical limit. Microalbuminuria was defined according to the recommendation of the American Diabetes Association and the National Kidney Foundation (300 > UACR ≥ 30 mg/g Cr) [9,10]. Individual salt intake was assessed by estimating the 24-hour urinary salt excretion of subjects and it was calculated using a previously reported formula [11].

Blood pressure was measured by a standard sphygmomanometer, after subjects were seated in a chair for 5 min with their backs supported as well as their arms supported at heart level. Proper cuff size was determined based on arm circumference. Three consecutive blood pressure measurements were taken, with two minutes between each measurement, and the mean of the second and third measurements was recorded as the blood pressure. Hypertension was defined as systolic blood pressure (SBP) ≥140 mmHg and/or diastolic blood pressure (DBP) ≥ 90 mmHg, or the use of antihypertensive medications. In this study, diabetes mellitus was defined as fasting plasma glucose ≥ 126 mg/dL or the use of anti-diabetic medications and dyslipidemia was defined as low-density lipoprotein-cholesterol (LDL-cholesterol) ≥140 mg/dL, high-density lipoprotein-cholesterol (HDL-cholesterol) < 40 mg/dL, triglyceride ≥ 150 mg/dL, or the use of antidyslipidemic medications [12]. ECGs were recorded after subjects had rested for 1 min in the supine position in an air-conditioned room.

### Statistical analysis

**Figure 1:**
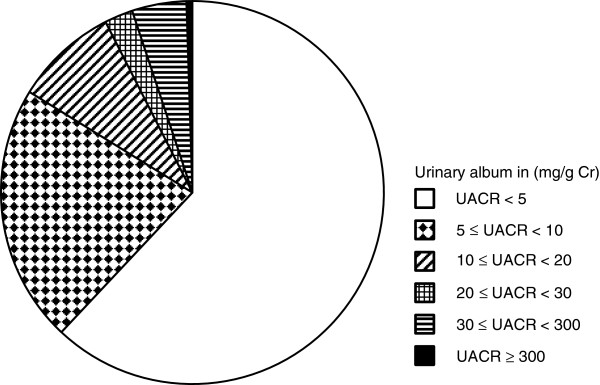
**Distribution of urinary levels of albumin.** Urinary albumin was detected in 7265 subjects (UACR ≥ 300 mg/g Cr, n = 41, 0.5%; 300 > UACR ≥ 30 mg/g Cr, n = 362, 4.6%; 30 > UACR ≥ 20 mg/g Cr, n =192, 2.4%; 20 > UACR ≥ 10 mg/g Cr, n = 694, 8.7%; 10 > UACR ≥ 5 mg/g Cr, n = 1737, 21.8%; UACR < 5 mg/g Cr, n = 4239, 53.2%). UACR indicates the ratio of the concentrations of urinary albumin to urinary creatinine.

All statistical analyses were performed using StatView 5.0 (SAS Institute, Inc., Cary, USA). Data in the text and the tables were expressed as mean ± standard deviation. Because the distribution of UACR was skewed rightward, these values were expressed as median ± absolute deviation and were log-transformed before statistical analysis. Differences between two groups were compared by unpaired Student’s *t* tests. A corrected chi-square test was used for comparisons between categorical data. Subjects were divided into six groups (Figure 1) according to their urinary level of albumin. The relationship between urinary excretion of albumin and blood pressure, fasting plasma glucose, or salt intake was investigated (ANOVA followed by Scheffe’s post-hoc analysis). Univariate and multivariate regression analyses were performed in order to investigate the relationship between urinary levels of albumin and other variables. A *P* value of less than 0.05 was considered to be statistically significant.

## Results

The characteristics of all study subjects are shown in Table 1, with 31.6% having hypertension, 7.4% having diabetes mellitus, and 44.1% having dyslipidemia. Of the subjects with these conditions, 66.3%, 67.9%, and 26.0%, respectively, were under medical treatment for the disorder. A total of 1642 subjects (20.6%) were overweight (body mass index (BMI) > 25 kg/m^2^. Urinary albumin was detected in most subjects (n = 7265, 91.2%). More than half of the subjects (53.2%), had minimal excretion levels of albumin (UACR < 5 mg/g Cr) and about 4.6% of subjects showed microalbuminuria (300 > UACR ≥ 30mg/g Cr) (Figure 1).

**Table 1:** Characteristics of subjects

	**Total (n = 7963)**	**Male (n = 5095)**	**Female (n = 2868)**
Age (years old)	56.2 ± 11.8	56.0 ± 11.9	55.6 ± 11.7
Waist circumference (cm)	83.3 ± 8.7	84.7 ± 8.3	81.0 ± 8.9
Body mass index (kg/m^2^)	22.7 ± 3.2	23.2 ± 3.0	21.9 ± 3.3
SBP (mmHg)	124.1 ± 15.1	125.6 ± 14.8	121.6 ± 15.5
DBP (mmHg)	76.3 ± 9.5	77.7 ± 9.4	73.8 ± 9.0
Serum creatinine (mg/dL)	0.76 ± 0.22	0.84 ± 0.24	0.62 ± 0.10
Uric acid (mg/dL)	5.5 ± 1.4	6.0 ± 1.3	4.5 ± 1.0
Fasting plasma glucose (mg/dL)	95.8 ± 17.9	98.1 ± 19.5	91.7 ± 13.6
LDL-cholesterol (mg/dL)	118.5 ± 26.9	118.5 ± 27.0	118.4 ± 26.7
HDL-cholesterol (mg/dL)	58.5 ± 13.8	55.6 ± 13.1	63.8 ± 13.5
Triglyceride (mg/dL)	107.8 ± 71.4	119.9 ± 80.7	86.2 ± 43.4
Urinary albumin (mg/g Cr)	3.89 ± 2.02	3.84 ± 2.13	3.94 ± 1.81
Estimated salt intake (g/day)	10.9 ± 3.4	12.3 ± 3.1	8.3 ± 2.1
Current smoking (n [%])	1692 [21.2%]	1547 [30.4%]	145 [5.1%]
ECG voltage^a^ (mV)	2.21 ± 0.93	2.33 ± 0.97	2.00 ± 0.82

Values are the mean ± standard deviation or the number of subjects, except for urine albumin (median ± absolute deviation). SBP indicates systolic blood pressure; DBP, diastolic blood pressure; LDL, low-density lipoprotein; HDL, high-density lipoprotein, Cr; creatinine.

^a^Electrocardiogram voltage (SV_1_ + RV_5_).

**Figure 2:**
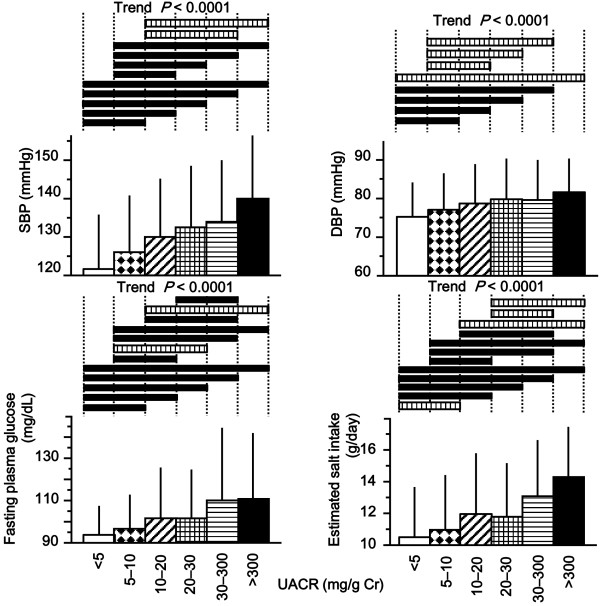
**The relationship of urinary albumin with blood pressure, plasma glucose, and salt intake (n=7963).** The solid and hatched horizontal bars in the upper part of the figure denote that a significant difference was determined by Scheffe’s post-hoc test with *P* < 0.001 or *P* < 0.05, respectively. UACR indicates the ratio of the concentrations of urinary albumin to urinary creatinine.

Regression analyses were performed in a subgroup of subjects with detectable urinary albumin (urinary albumin ≥ 1.1 mg/L; n = 7265, 91.2%). Using univariate regression analysis, UACR was correlated with age, male gender, waist circumference, BMI, SBP, serum creatinine, uric acid, fasting plasma glucose, dyslipidemia, salt intake, ECG voltage, and use of inhibitors of the renin-angiotensin system (Table 2). Age, male gender, SBP, serum creatinine, fasting plasma glucose, salt intake, smoking status, ECG voltage, and use of inhibitors of the renin-angiotensin system were independent predictors of urinary albumin among the variables that show close correlation with urinary excretion of albumin after adjustment for possible factors listed in Table 3. Similar results were obtained in an analysis where all subjects (n = 7963) were included in the regression model that used log transformed (UACR + 0.5) as a dependent variable. Age (coefficient 0.113, *P* < 0.0001), male gender (−0.154, *P* < 0.0001), SBP (0.139, *P* < 0.0001), serum creatinine (0.125, *P* < 0.0001), salt intake (0.105, *P* < 0.0001), smoking (0.124, *P* < 0.0001), ECG voltage (0.056, *P* < 0.0001), and use of inhibitors of the renin-angiotensin system (0.038, *P* < 0.001) independently correlated with UACR after adjustment for waist circumference, uric acid, fasting plasma glucose, and dyslipidemia (*R*^*2*^ = 0.111, *F* = 83.0, *P* < 0.0001). In line with these observations, SBP, DBP, fasting plasma glucose, and salt intake were observed to increase with increasing urinary excretion of albumin in analyses that included all subjects (Figure 2). These results were further supported by a multivariate logistic regression analysis where risks of microalbuminuria were calculated (Table 4).

**Table 2:** Results of univariate analyses demonstrating the relationship between urinary albumin and other variable

	**Standardized coefficient**	***P *value**
Age	0.251	< 0.0001
Gender; male	0.040	< 0.001
Waist circumference	0.120	< 0.0001
Body mass index	0.076	<0.0001
SBP	0.243	< 0.0001
Serum creatinine	0.116	< 0.0001
Uric acid	0.043	< 0.001
Fasting plasma glucose	0.221	< 0.0001
LDL-cholesterol	−0.006	0.61
HDL-cholesterol	−0.057	< 0.0001
Triglyceride	0.075	< 0.0001
Dyslipidemia	0.077	< 0.0001
Estimated salt intake	0.209	< 0.0001
Current smoking	0.015	0.21
ECG voltage^a^	0.099	< 0.0001
RAS inhibitors	0.145	< 0.0001

Univariate analysis was conducted in subjects with detectable urinary albumin (n=7265). SBP indicates systolic blood pressure; LDL, low-density lipoprotein; HDL, high-density lipoprotein; RAS, renin-angiotensin system.

^a^Electrocardiogram voltage (SV_1_ + RV_5_).

**Table 3:** Multivariate regression analysis demonstrates the relationship of urinary excretion of albumin to other variables

	***R***^***2 ***^**= 0.163, *F *= 117.4, *P *< 0.0001**	
	**Standardized coefficient**	***P *value**
Age	0.152	< 0.0001
Gender; male	−0.215	< 0.0001
Waist circumference	−0.017	0.16
SBP	0.130	< 0.0001
Serum creatinine	0.135	< 0.0001
Uric acid	−0.004	0.76
Fasting plasma glucose	0.155	< 0.0001
Dyslipidemia	0.006	0.59
Estimated salt intake	0.185	< 0.0001
Current smoking	0.103	< 0.0001
ECG voltage^a^	0.055	< 0.0001
RAS inhibitors	0.054	< 0.0001

Analysis in subjects with detectable urinary albumin (n = 7265).

SBP indicates systolic blood pressure; RAS, renin-angiotensin system.

^a^Electrocardiogram voltage (SV_1_ + RV_5_).

**Table 4:** Results of logistic regression analysis demonstrating a risk of microalbuminuria

	**Odd’s ratio (95%CI)**	***P *value**
Age (years)	1.047 (1.035–1.059)	< 0.0001
Gender; male	0.467 (0.324–0.671)	< 0.0001
Waist circumference (cm)	1.010 (0.996–1.024)	0.16
SBP (mmHg)	1.027 (1.020–1.035)	< 0.0001
Serum creatinine (mg/dL)	7.090 (3.863–13.015)	< 0.0001
Uric acid (mg/dL)	1.081(0.983–1.188)	0.11
Fasting plasma glucose (mg/dL)	1.019 (1.015–1.023)	< 0.0001
Dyslipidemia	1.132 (0.908–1.412)	0.27
Estimated salt intake (g/day)	1.142 (1.103–1.183)	< 0.0001
Current smoking	2.040 (1.553–2.681)	< 0.0001
ECG voltage^a^	1.151 (1.030–1.285)	< 0.05
RAS inhibitors	1.485 (1.150–1.917)	< 0.01

Logistic analysis in all subjects (n = 7963).

SBP indicates systolic blood pressure; RAS, renin-angiotensin system.

^a^Electrocardiogram voltage (SV_1_ + RV_5_).

## Discussion

This study demonstrates that the majority of the Japanese general population has detectable albuminuria but only 4.6% present with abnormal microalbuminuria. There is a close association between urinary excretion of albumin, even in normal range, and cardiovascular morbidity and mortality. This association suggests that an approach of reducing urinary albumin could be used to prevent cardiovascular disease, which is a leading cause of death. Lifestyle modification is one possible mechanism to improve urinary excretion of albumin because both salt intake and SBP independently correlate with urinary albumin.

Several studies have reported on the prevalence of microalbuminuria in the general population including 7% of subjects in the PREVEND study, and 3.8% of subjects in the INTERMAP study. The prevalence of subjects with microalbuminuria in those studies are similar to the present study [13,14]. In contrast, the Takahata study reported that the prevalence of microalbuminuria was 13.7% in the general population [15]. We observed that the excretion of urinary albumin is closely associated with age, male gender, blood pressure, salt intake, and various other factors. Thus, the discrepancy in the prevalence of microalbuminuria between the present study and previous studies may be attributable to differences in the characteristics of the subjects studied. Indeed, the mean age was older and the blood pressure was higher in the Takahata study than in the present study. A linear correlation of urinary excretion of albumin with future cardiovascular and non-cardiovascular death was observed in the PREVEND study and the adjusted hazard of major cardiovascular events was increased by 5.9% for every 0.4 mg/mmol increase in UACR levels in the HOPE trial [1,7]. These studies demonstrate that even small increases in urinary excretion of albumin within the ‘normal level’ can increase the risk of future cardiovascular events. Furthermore, a reduction in urinary albumin levels is associated with a reduction in cardiovascular events [16,17]. These observations suggest that urinary excretion of albumin may be a surrogate marker that will useful for the evaluation of individual risk in clinical settings. The findings of others in combination with the findings of this study indicate that many people may benefit from intensive intervention to reduce or normalize the urinary excretion of albumin.

The beneficial effects of inhibitors of the renin-angiotensin system on urinary albumin have already been established [13,18-20]. However, in most cases medical intervention is not realistic because of the wide prevalence of albuminuria, including ‘normal’ albuminuria, in the general population. A public health approach aimed at lifestyle modification that includes restriction of salt intake and high calorie diet, may be effective, because of the observed close relationship between urinary excretion of albumin and salt intake, SBP, and fasting plasma glucose.

Although the present study does not establish a causal relationship between the urinary excretion of albumin with other factors, several mechanisms underlying the observed relationship can be proposed. The kidney is an important target organ of hypertension and the relationship between increased blood pressure and albuminuria has been established. Excess salt intake produces an excess volume load and, thereby, causes an increase in intra-glomerular pressure and glomerular hyperfiltration, which results in urinary excretion of albumin. An increase in blood pressure is also involved in the mechanism underlying salt-induced albuminuria. An elevated fasting plasma glucose level indicates the presence of insulin resistance that promotes sodium re-absorption and may increase the volume load. Alternatively, endothelial dysfunction may be involved in the mechanism underlying the observed relationship between urinary excretion of albumin and other factors [21-23]. Many factors that have a close association with the urinary excretion of albumin in the present study also promote atherosclerosis. Since endothelial dysfunction is an early step of atherosclerosis, these factors may cause urinary albumin excretion via endothelial damage [24]. Indeed, in the Hoorn study, a linear association of microalbuminuria with endothelium function as assessed by flow-mediated vasodilation was observed in subjects either with or without diabetes [22]. Furthermore, inhibition of the endothelial nitric oxide pathway causes albuminuria [23]. The positive correlation between the use of inhibitors of the renin-angiotensin system and urinary albumin may have been attributable to high prevalence of microalbuminuria among hypertensive patients as compared to normotensive subjects. Alternatively, patients with albuminuria may have been preferably prescribed with renin-angiotensin system inhibitors though the prevalence of microalbuminuria was not significantly different between hypertensive patients with and without renin-angiotensin system inhibitors (data not shown).

The interpretation of the present study is limited by the following conditions. Firstly, the urinary excretion of albumin was measured only once. Secondly, study subjects were participants in our health checkup program, which could introduce a selection bias. Thirdly, clinical significance of albuminuria as a cardiovascular risk was not assessed in the present study. Finally, a causal relationship cannot be established because this was a cross-sectional study. Longitudinal follow-up studies are necessary to draw a definite conclusion.

## Conclusion

The majority of the general population has detectable urinary excretion of albumin and 4.6% show microalbuminuria. The urinary excretion of albumin was shown to be closely associated with salt intake as well as blood pressure. These data indicate the importance of dietary salt restriction for the prevention of cardiovascular disease as well as end-stage renal disease.

## Abbreviations

UACR: The ratio of urinary albumin to creatinine concentrations; Cr: Creatinine; ECG: Electrocardiogram; SBP: Systolic blood pressure; DBP: Diastolic blood pressure; LDL-cholesterol: Low-density lipoprotein-cholesterol; HDL-cholesterol: High-density lipoprotein-cholesterol.

## Competing interests

The authors declare that they have no competing interests.

## Authors’ contributions

ST analyzed data and mainly drafted the manuscript. HT participated in the design of the study, substantially contributed to the acquisition of data. YD substantially designed the study, substantially contributed to the interpretation of the data, and critically revised the manuscript. GK participated in the design of the study and contributed to the interpretation of the data. All the authors have read and approved the final manuscript.
